# Validity and Reliability of A-Mode Ultrasound for Body Composition Assessment of NCAA Division I Athletes

**DOI:** 10.1371/journal.pone.0153146

**Published:** 2016-04-13

**Authors:** Dale R. Wagner, Dustin L. Cain, Nicolas W. Clark

**Affiliations:** Human Movement Science Program, Utah State University, Logan, Utah, United States of America; Sonoma State University, UNITED STATES

## Abstract

This study evaluated the validity and reliability of the BodyMetrix™ BX2000 A-mode ultrasound for estimating percent body fat (%BF) in athletes by comparing it to skinfolds and the BOD POD. Forty-five (22 males, 23 females) National Collegiate Athletic Association (NCAA) Division-I athletes volunteered for this study. Subjects were measured once in the BOD POD then twice by two technicians for skinfolds and ultrasound. A one-way repeated-measures ANOVA revealed significant differences between body composition methods (*F* = 13.24, *p* < 0.01, η² = 0.24). This difference was further explained by a sex-specific effect such that the mean difference between ultrasound and BOD POD was large for females (~ 5% BF) but small for males (~ 1.5% BF). Linear regression using the %BF estimate from ultrasound to predict %BF from BOD POD resulted in an *R*^2^ = 0.849, *SEE* = 2.6% BF and a *TE* = 4.4% BF. The inter-rater intraclass correlation *(ICC)* for skinfold was 0.966 with a large 95% confidence interval (CI) of 0.328 to 0.991. The inter-rater *ICC* for ultrasound was 0.987 with a much smaller 95% CI of 0.976 to 0.993. Both skinfolds and ultrasound had test-retest *ICC*s ≥ 0.996. The BX2000 ultrasound device had excellent test-retest reliability, and its inter-rater reliability was superior to the skinfold method. The validity of this method is questionable, particularly for female athletes. However, due to its excellent reliability, coaches and trainers should consider this portable and easy to use A-mode ultrasound to assess body composition changes in athletes.

## Introduction

Athletes strive for a competitive advantage, and for many athletes building a lean body with a low body fat percentage (%BF) can help them achieve a higher level of performance. This is particularly the case in: (a) gravitational sports in which a high body mass hinders performance, (b) aesthetic sports in which there is a perceived ideal shape, and (c) weight class sports in which competition is organized into categories of body mass [1]. With that in mind, some athletes put themselves at risk of health problems through extreme dieting, disordered eating, and fluid restriction in an effort to achieve a particular weight or %BF [1]. Whether it be tracking fat loss results or monitoring healthy habits, body composition assessment can serve as a beneficial tool for both coaches and athletes to help maximize performance and estimate a healthy competitive weight.

Various body composition testing methods are used in sports medicine and exercise science. The most commonly used criterion or reference methods thought to be the most valid include dual-energy X-ray absorptiometry (DXA), hydrodensitometry, and air displacement plethysmography (ADP), more commonly known as the BOD POD [2]. However, these large laboratory devices are expensive, not always practical, and in the case of DXA require specialized personnel or training to operate. Small, portable field methods such as bioelectrical impedance (BIA) and skinfolds offer more flexibility of measurement and could be advantageous when measuring athletes at event-site locations. However, the validity of these field methods is less than the reference methods [2].

A possible alternative that is small enough to be a portable field method yet potentially as accurate as the laboratory methods is ultrasound. The technical principles of this method as well as the strengths and limitations of ultrasound to provide reliable and valid body composition assessments were recently reviewed [3]. The review concluded that ultrasound has great potential to provide reliable and accurate estimates of subcutaneous fat, but more research is needed on new devices and software that were designed specifically for the purpose of body composition assessment. Additionally, an Ad Hoc Working Group on Body Composition, Health and Performance of the International Olympic Committee Medical Commission recently suggested that ultrasound and the emerging software and technological advances of this method might offer advantages over other methods for assessing the body composition of Olympic athletes [4]. This research team developed software that can accurately assess subcutaneous adipose tissue to within 0.1 to 0.5 mm of thickness [5]. However, the software is meant to be used with a high-resolution B-mode ultrasound unit. These medical devices are costly (typically > $20,000), and the software is > $2,000.

A novel and relatively inexpensive (< $2,000) A-mode ultrasound device (BodyMetrix™ BX2000), with user-friendly body composition-specific software, is now commercially available. This device was mentioned in a review of ultrasound for body composition assessment, and it was noted that there are only a few published validity studies on this ultrasound [3]. If found to be accurate, it would provide a lower cost alternative to the B-mode ultrasound suggested by Müller et al. [5] for assessing the body composition of elite athletes, and greater portability than the laboratory methods. In the hands of a skilled technician, the skinfold method is the preferred field method for estimating the %BF of athletes [6]. The BX2000 is marketed as an alternative to the skinfold method, and the same measurement sites for common skinfold equations are programmed into the software that accompanies this ultrasound device. Thus, the purpose of this study was to test the validity, test-retest reliability, and inter-rater reliability of the BX2000 A-mode ultrasound by comparing it to skinfolds and the BOD POD in a sample of Division-I collegiate athletes.

## Materials and Methods

### Ethics statement

This study was approved by the Institutional Review Board of Utah State University (protocol #6239). All participants were informed of the benefits and risks of the investigation prior to signing an informed consent document.

### Design

This was a repeated-measures design such that all athletes in the study had their body composition assessed by all three methods in a single session. Two experienced technicians took all of the skinfold and ultrasound measurements separately, in duplicate, in order to evaluate test-retest reliability and inter-rater reliability. Additionally, a third technician was responsible for BOD POD measurements only. The technicians were blinded to each other’s results.

### Subjects

Forty-five (22 males, 23 females) National Collegiate Athletic Association (NCAA) Division-I athletes were recruited to participate in this study. The descriptive statistics are represented in Table 1. The student-athletes were from 7 different sports: football (*n* = 8), golf (*n* = 7), women’s gymnastics (*n* = 9), softball (*n* = 4), women’s volleyball (*n* = 1), and men’s (*n* = 7) and women’s tennis (*n* = 9).

**Table 1:** Descriptive statistics (mean ± SD).

	Total (*N* = 45)	Male (*n* = 22)	Female (*n* = 23)
Age (years)	20.1 ± 1.6	20.6 ± 1.6	19.6 ± 1.4
Height (cm)	172.2 ± 10.2	179.8 ± 6.5	165.0 ± 7.3
Weight (kg)	71.8 ± 12.4	80.8 ± 10.7	63.3 ± 6.6
BMI (kg/m²)	24.1 ± 2.4	24.9 ± 2.4	23.3 ± 2.2

### Preliminary procedures

Following informed consent and prior to body composition testing, participants were asked to void their bladder and bowels. Height was measured to the nearest 1 mm using a wall-mounted stadiometer (Seca 216, Seca Corp., Ontario, CA). Weight was measured to the nearest 0.01 g during the BOD POD procedure. The BOD POD (Cosmed USA, Inc., Concord, CA) was calibrated following the manufacturer’s guidelines, and the precision of the skinfold caliper (Lange, Cambridge Scientific Industries, Inc., Cambridge, MD) was checked against 15 mm and 25 mm calibration blocks.

### BOD POD

Participants were measured while wearing tight-fitting clothing (e.g., lycra swimsuit or compression clothing) according to standardized procedures [6], and manufacturer’s guidelines were followed for the BOD POD assessment. Thoracic gas volume (TGV) was measured to attain the highest degree of precision possible with the BOD POD measurement. The same technician performed all of the BOD POD tests.

### Skinfold

Following the BOD POD assessment, participants underwent the skinfold and ultrasound assessments. The Jackson and Pollock 3-site skinfold locations and equations [7,8] were used to estimate %BF. The sites included the chest, abdomen, and thigh for males [7], and the triceps, suprailiac, and thigh for females [8]. The same sites were used for the ultrasound measurements. The sites were marked by technician 1 using a surgical marker to maintain consistency for the ultrasound measurements. Standardized procedures for the skinfold technique as described by Heyward and Wagner [6] were followed. Two technicians performed the measurements in rotational order and then repeated the measurement rotation such that there were two sets of measurements for each technician. They were blinded to each other’s readings. Both technicians were experienced with the skinfold technique; however, the length of experience varied with technician 1 having 20 y of experience and technician 2 having 6 y of experience.

### Ultrasound

The BodyMetrix™ BX2000 (IntelaMetrix, Inc., Livermore, CA) 2.5 MHz, A-mode ultrasound in conjunction with the associated Body View Professional software (IntelaMetrix, Inc., Livermore, CA) was used to make the ultrasound measurements. The software prompts the technician to select an “athletic type” from one of three choices: elite, athletic, non-athletic. Elite, defined by the manufacturer as “individuals that generally have good muscle definition and little excess fat,” was used as the default setting for this sample. The manufacturer’s recommendations for making single-point ultrasound measurements at the previously mentioned skinfold sites were followed. This included placing conducting gel on both the ultrasound transducer head and the measurement site on the participant to minimize friction and allow the transducer to freely move on the participant’s skin. The transducer was then moved back and forth about a quarter inch (0.64 cm) to either side of the measurement site for about 3 seconds. Care was taken to minimize the pressure applied to the transducer head so as not to compress the skin, thereby altering the subcutaneous fat thickness. Subcutaneous adipose tissue thickness was recorded at each measurement site, and the %BF was automatically calculated from the Body View Professional software. The same technicians that performed the skinfold measurements also took the ultrasound measurements, again being blinded to the other’s readings. As with the skinfold measurements, both technicians took the ultrasound measurements in a rotational order and then repeated the rotation, thereby both technicians obtained two readings of each measurement site. Both technicians had about 6 months of experience with the ultrasound device.

### Estimates of %BF

The Body View Professional software uses a proprietary algorithm to convert the subcutaneous fat thicknesses obtained from the BodyMetrix™ BX2000 A-mode ultrasound to a %BF value (D. Watts, personal communication, 2015). A value for body density (Db) is not provided. Thus, in order to make comparisons among the three methods, the Db values from the BOD POD and skinfolds were converted to %BF values using the conversion formula of Siri [9]. Although other conversion formulas are recommended for converting the Db of females and non-Caucasians to %BF [6], the Siri [9] formula was used for all athletes. This was done for consistency and because no Db conversion formula or sex-specific or ethnic-specific correction factor is provided for the BX2000 ultrasound.

### Statistical analyses

All data were analyzed using SPSS version 22 (IBM, Inc., Armonk, NY). Statistical significance was accepted at *p* < 0.05. Means and standard deviations were calculated for all variables, and normality of sample distribution was assessed with the Shapiro-Wilk test. Both the test-retest reliability and inter-rater reliability of %BF estimated from skinfolds and ultrasound were assessed with intraclass correlation (*ICC*_3,2_) with a two-way mixed average measures model and absolute agreement. Additionally, the standard error of measurement [*SEM* = *SD*√(1-*ICC*)] was calculated in order to obtain the minimal difference (*MD* = *SEM* x 1.96 x √2) for the test-retest reliability of the skinfold and ultrasound methods. Values greater than the *MD* for repeat measurements, in weight change studies for example, are considered to be “real” changes that exceed the error of measurement [10]. The relationship between skinfold thickness and uncompressed subcutaneous adipose tissue thickness from ultrasound at each of the measured sites was evaluated with Pearson correlation. A one-way repeated-measures analysis of variance (ANOVA) was used to compare mean differences between the %BF estimates of the 3 assessments (BOD POD vs skinfold vs ultrasound) with sex as a covariate, and Sidak post-hoc was used to further elucidate the differences from the ANOVA. Other validity criteria were also used such as evaluating the magnitude of the standard error of estimate (*SEE*), or the average deviation of individual scores around the line of best fit, and total error (*TE*), or the average deviation of individual scores from the line of identity, as described by Heyward and Wagner [6]. Bland-Altman [11] plots were also used to evaluate individual differences rather than only mean differences.

## Results

Twelve of the 45 subjects were unable to get a valid measured TGV even after 5 attempts; thus their predicted TGV was used. Research suggests that the difference in predicted TGV is not significantly different from measured TGV for the majority of athletes [12].

Statistical assumptions were tested on BOD POD data before the repeated-measures ANOVA was run. There were no outliers, and data were normally distributed (Shapiro-Wilk *p* = 0.31). Mauchly’s test of sphericity was significant (*p* < 0.01), so the Greenhouse-Geisser method was used to evaluate the within-subject effects.

The test-retest *ICC* for skinfold and ultrasound for technician 1 was 0.999 (95% CI = 0.999–1.000) and 0.996 (95% CI = 0.993–0.998), respectively. Similarly, for technician 2 the *ICC* was 0.996 (95% CI = 0.993–0.998) for skinfold and 0.993 (95% CI = 0.987–0.996) for ultrasound. The *MDs* for the skinfold method were 0.7% BF and 1.5% BF for technician 1 and 2, respectively. The *MD* for the ultrasound method was 1.3% BF for technician 1 and 1.8% BF for technician 2. The inter-rater *ICC* for skinfold was 0.966, but with a large 95% CI of 0.328 to 0.991. The inter-rater *ICC* for ultrasound was 0.987, but with a much smaller 95% CI of 0.976 to 0.993.

The skinfold and subcutaneous fat from the ultrasound were highly correlated at each of the measurement sites (*r* > 0.68, *p* < 0.01). Also, with the exception of the suprailiac site, the correlations for technician 1 were slightly higher than for technician 2. The skinfold-ultrasound correlation for each site from both technicians can be found in Table 2. The skinfold and ultrasound measurements for each site from both technicians are in Table 3.

**Table 2:** Correlation between skinfolds and ultrasound at each measurement site, for each trial, by both technicians.

	Males (*n* = 22)						Females (*n* = 23)					
	Chest		Abdomen		Thigh		Triceps		Suprailiac		Thigh	
Trial	1	2	1	2	1	2	1	2	1	2	1	2
Tech1	.809	.847	.817	.776	.938	.943	.854	.824	.786	.840	.745	.897
Tech2	.801	.731	.810	.798	.866	.920	.820	.687	.822	.906	.698	.769

**Table 3:** Means ± SD (mm) for each skinfold and ultrasound measurement site, for each trial, measured by each technician.

Trial	1	2	1	2
	Males (*n* = 22)			
	Chest Skinfold		Chest Ultrasound	
Tech 1	6.3 ± 2.3	6.2 ± 2.2	4.8 ± 1.3	4.9 ± 1.4
Tech 2	9.1 ± 3.2	8.8 ± 3.1	4.9 ± 1.5	4.7 ± 1.4
	Abdomen Skinfold		Abdomen Ultrasound	
Tech 1	16.4 ± 6.6	16.2 ± 6.8	11.5 ± 4.7	11.3 ± 4.9
Tech 2	18.7 ± 7.7	18.3 ± 7.6	11.2 ± 5.0	11.3 ± 5.1
	Thigh Skinfold		Thigh Ultrasound	
Tech 1	10.2 ± 2.9	10.1 ± 2.9	5.2 ± 1.2	5.1 ± 1.3
Tech 2	11.4 ± 3.4	11.8 ± 3.4	5.6 ± 3.1	5.1 ± 1.3
	Females (*n* = 23)			
	Triceps Skinfold		Triceps Ultrasound	
Tech 1	16.4 ± 5.1	16.3 ± 5.4	9.6 ± 2.8	9.8 ± 2.9
Tech 2	20.3 ± 5.3	20.5 ± 5.5	10.0 ± 2.8	9.7 ± 3.0
	Suprailiac Skinfold		Suprailiac Ultrasound	
Tech 1	18.5 ± 5.9	19.1 ± 6.4	11.8 ± 3.6	12.1 ± 4.4
Tech 2	23.0 ± 7.6	23.5 ± 8.4	11.6 ± 4.5	11.3 ± 3.8
	Thigh Skinfold		Thigh Ultrasound	
Tech 1	23.0 ± 6.3	22.7 ± 6.3	10.4 ± 2.8	10.7 ± 2.6
Tech 2	25.0 ± 5.9	24.3 ± 5.9	10.2 ± 2.3	10.6 ± 2.4

The results for the ANOVA indicated a statistically significant difference between body composition methods, *F* = 13.24, *p* < 0.01, η² = 0.24. The interaction of body composition method with sex was also significant (*F* = 14.68, p < 0.01, η² = 0.25). Inspection of these data show that there is reasonably good agreement across technicians and methods for the male athletes, but not for females. Mean %BF data for the three methods are in Table 4.

**Table 4:** Body fat percentages (%BF) from the three methods (mean ± SD) for each trial by each technician.

	Skinfold	Ultrasound	Bod Pod
Technician 1; Trial 1	Total: 15.9 ± 8.1	Total: 18.2 ± 7.6	Total: 14.9 ± 6.7
	Male: 8.9 ± 3.2	Male: 11.7 ± 3.4	Male: 10.1 ± 3.9
	Female: 22.6 ± 5.0	Female: 24.3 ± 4.7	Female: 19.6 ± 5.5
Technician 1; Trial 2	Total: 15.8 ± 8.2	Total: 18.3 ± 7.9	
	Male: 8.8 ± 3.2	Male: 11.7 ± 3.6	
	Female: 22.6 ± 5.2	Female: 24.7 ± 5.0	
Technician 2; Trial 1	Total: 18.5 ± 8.7	Total: 18.1 ± 7.7	
	Male: 10.8 ± 3.5	Male: 11.4 ± 3.6	
	Female: 25.9 ± 4.9	Female: 24.4 ± 4.5	
Technician 2; Trial 2	Total: 18.5 ± 8.8	Total: 18.0 ± 7.7	
	Male: 10.7 ± 3.5	Male: 11.5 ± 3.9	
	Female: 25.9 ± 5.1	Female: 24.2 ± 4.6	

The Sidak post-hoc analysis revealed that the %BF estimations from the two skinfold trials of technician 1 were similar to each other (*p* = 1.00) and not significantly different than the BOD POD (*p* = 0.50 and 0.62 for trial 1 and 2, respectively). However, these values were significantly less than the %BF estimations from technician 2’s skinfolds (p < 0.01). All of the %BF estimates from the ultrasound measurements were significantly greater than the estimates from the BOD POD and technician 1’s skinfolds (*p* < 0.01). The difference between the two technicians for the ultrasound was not significant (*p* = 0.92 to *p* = 1.00). Technician 2’s skinfolds were similar to each other (*p* = 1.00) and nearly identical to his ultrasound measurements (*p* = 1.00).

**Fig 1:**
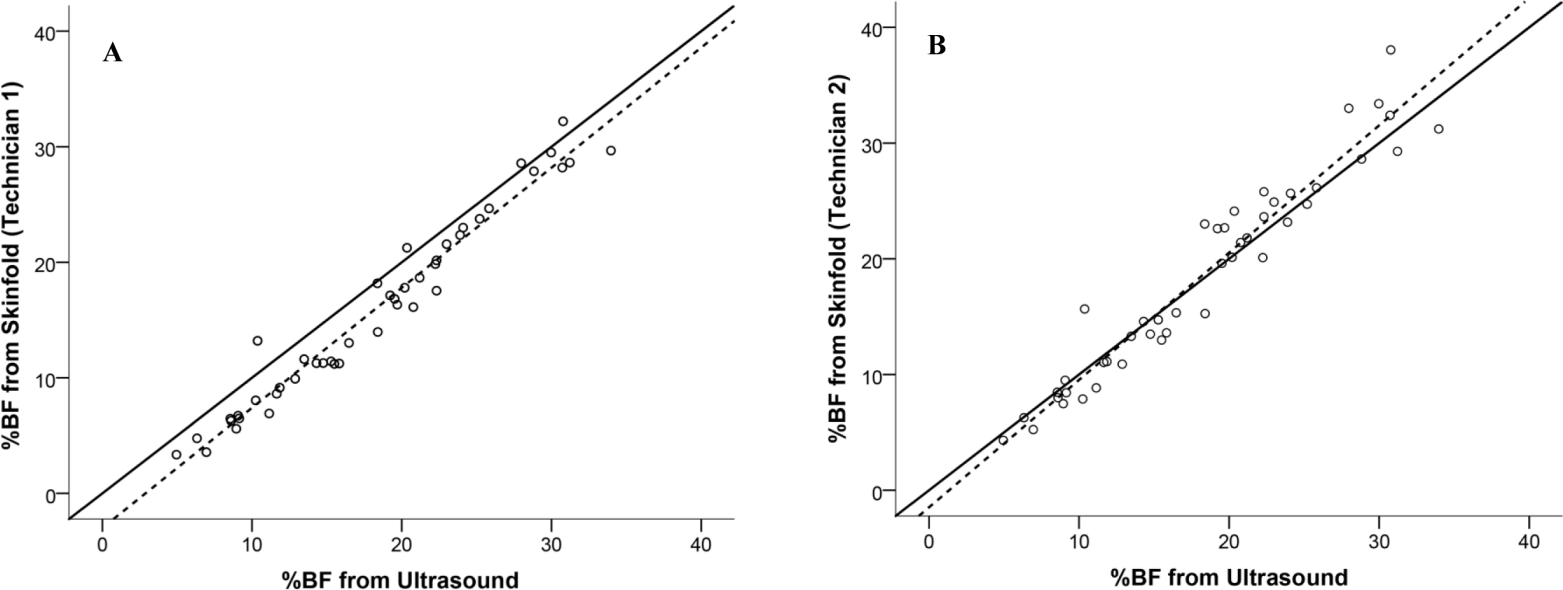
**Relationship between estimated body fat percentage (%BF) from ultrasound and skinfold for technician 1 (a) and technician 2 (b).** Solid line represents the line of identity and dashed line is the regression line.

**Fig 2:**
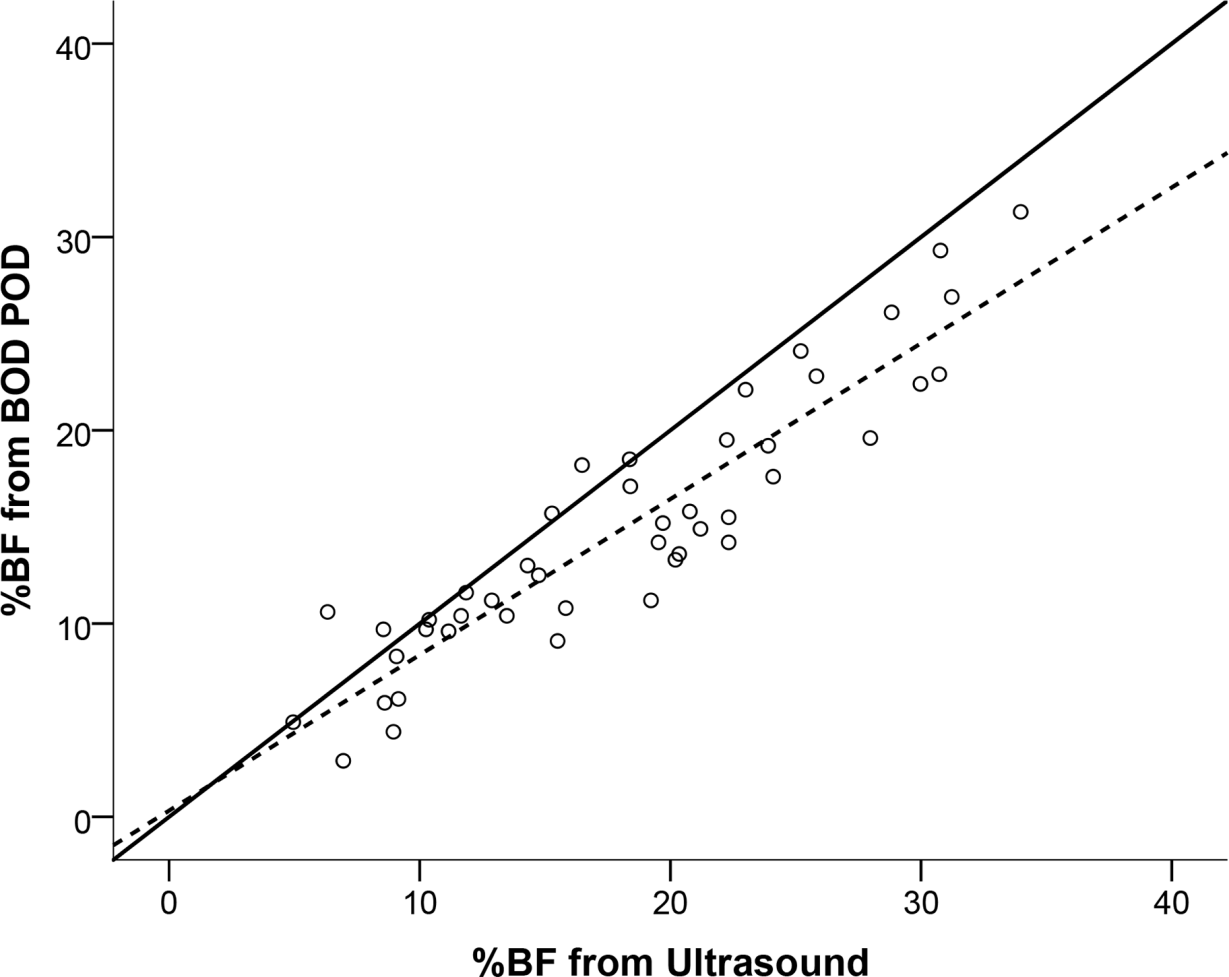
Relationship between estimated body fat percentage (%BF) from ultrasound and from the Bod Pod. Solid line represents the line of identity and dashed line is the regression line.

**Fig 3:**
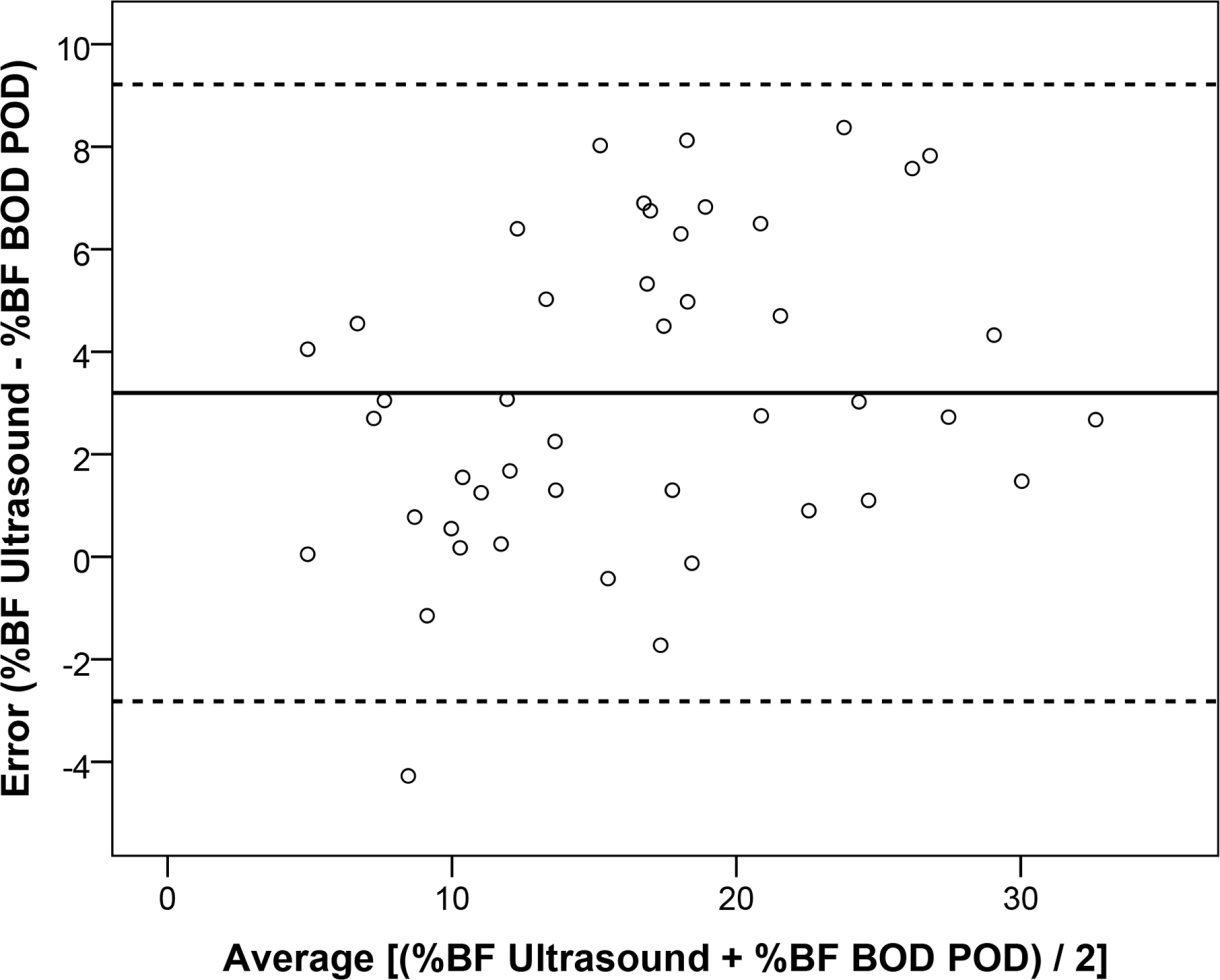
Bland-Altman analysis of the residual scores. Solid line is the constant error and the dashed lines are ± 2 SD.

All four of the %BF estimates from the ultrasound (two trials from two technicians) were similar and not significantly different; thus they were averaged and compared to the %BF estimates from the skinfold measurements of technician 1 (Fig 1A), technician 2 (Fig 1B), and the BOD POD (Fig 2). Linear regression using the %BF estimate from ultrasound to predict %BF from the BOD POD resulted in an *R*^2^ = 0.849, *SEE* = 2.6% BF and a *TE* = 4.4% BF. Finally, the Bland-Altman plot depicting individual errors for %BF estimated from ultrasound compared to the BOD POD is presented in Fig 3.

## Discussion

The purpose of this study was to test the validity and reliability of the BodyMetrix™ BX2000 A-mode ultrasound for estimating %BF in athletes by comparing it to skinfolds and the BOD POD. First, both the ultrasound and skinfolds had very high test-retest reliability which means both technicians were consistent with themselves. Aandstad and colleagues [13] recently reported test-retest *ICC*s ranging from 0.88 to 0.98 for %BF estimated from a variety of skinfold equations in a sample of military personnel. Despite high *ICC*s, they noted that six out of seven skinfold site measurements were significantly higher on the retest and that the limits of agreement were slightly wider for the skinfold method than BIA. For the BX2000 ultrasound, Smith-Ryan, et al. [14] reported an *ICC* of 0.98 and *MD* of 4.3% BF for a seven-site measurement, and Loenneke et al. [15] reported an *ICC* of 0.94 with an *MD* of 5.8% BF using the same three measurement sites performed in the present study. In contrast, the *MD*s for both technicians in the present study were < 2% BF for both the skinfold and ultrasound methods. Some potential reasons for superior test-retest reliability values for our study could be a leaner sample and a shorter time period between the first and second measurement. Our study sample consisted of relatively lean athletes, but Smith-Ryan et al. [14] measured overweight and obese adults. Both Loenneke et al. [15] and Smith-Ryan et al. [14] took their retest measurements on a different day (day-to-day reliability), whereas our repeat measurement was taken during the same session. Furthermore, we marked our measurement locations, which likely improved the reliability [6].

To the best of our knowledge, this is the first study to examine the inter-rater reliability of this A-mode ultrasound device for estimating %BF. The inter-rater *ICC*s were very large for both the skinfold and ultrasound methods; however, the 95% CI was very large for skinfolds and very narrow for ultrasound. This indicates that for the skinfold method the technicians were consistently inconsistent with technician 1 consistently recording lower skinfold measurements then technician 2. Previous researchers have also commented on the difficulty of obtaining high inter-rater reliability using the skinfold method [16,17]. Kispert and Merrifield [16] examined the inter-rater reliability of skinfolds by comparing the results of eight different raters who each measured three anatomical sites on 20 subjects. They concluded that the inter-rater reliability using the skinfold technique was insufficient for tracking body fat measurements. In the present study, the post-hoc analysis from the ANOVA confirmed a significant technician difference for skinfolds but no difference for the ultrasound measurements. On average, the two technicians’ skinfold measurements differed by about 1.9% BF on the male athletes and 3.3% BF on the female athletes (Table 4). In contrast, they differed by only about 0.2% BF when using the ultrasound method, regardless of sex being tested (Table 4).

We also considered the relationship between skinfold and ultrasound at the individual measurement sites (Table 2). Ulbricht, et al. [18] pointed out that the values for skinfold were greater than the values for ultrasound at any given site in their sample of military personnel. We also found this to be the case (Table 3). This is to be expected because a skinfold involves a double layer of skin along with the compressed fold of subcutaneous fat [6], whereas the ultrasound method is directly measuring the subcutaneous fat thickness [3]. Despite the difference in absolute value, it is logical to assume a high correlation between methods because they are both measuring subcutaneous fat. Surprisingly, Ulbricht et al. [18] reported weak, non-significant correlations between skinfold and ultrasound for about half of the nine sites that they measured. In contrast, correlation coefficients were > 0.70 at nearly every site for both sexes as measured by both technicians in the present study (Table 2). We cannot explain the low correlations reported by Ulbricht et al. [18]; however, the fact that the anatomical locations remained marked between the skinfold and ultrasound measurements likely contributed to our high correlations.

Regarding validity, there was a significant difference in the %BF estimates from the three methods such that the mean %BF from the BOD POD was about 3% lower than the mean %BF from the ultrasound, with the skinfold estimate of technician 1 matching closely to the BOD POD and the skinfold estimate of technician 2 matching closely to the ultrasound (Table 4). Assuming that the BOD POD is a valid criterion measure, the *SEE* of ultrasound was near the excellent category but the *TE* was only fair according to the subjective evaluative ratings reported by Lohman [19]. Upon closer inspection, sex was an important covariate. When only males were considered in the analysis, all differences to the BOD POD became non-significant, and both the skinfold and ultrasound methods were within ± 1.5% BF of the BOD POD. Additionally, the *TE* was reduced to 2.8% BF, a very good rating [19]. However, the BOD POD produced significantly lower %BF values than either the skinfolds or the ultrasound for females, and these were large mean differences ranging from 3.0% BF to 5.1% BF. Furthermore, the *TE* grew to 5.5% BF for the female only sample. In summary, There was good agreement between all three methods for the male athletes, but both the skinfold and ultrasound methods produced substantially higher %BF estimations than the BOD POD for female athletes.

Several other research teams have also done validity studies using portable A-mode ultrasound to estimate %BF with variable results. Pineau, et al. [20] used a combination of anthropometric dimensions and ultrasound measurements at the abdomen and mid-thigh to develop a new model to predict fat mass in 89 adults ranging in age from 18–60 y. They evaluated this against DXA, ADP, and BIA. The ultrasound estimates of %BF provided higher correlations to the DXA measurement than the ADP or BIA methods. The 95% limit of agreement was also narrower for ultrasound than ADP and BIA. This research team repeated their ultrasound model on a sample of 93 athletes [21]. They reported very high correlations to DXA for both males (r = 0.98) and females (r = 0.97) and an excellent 95% limit of agreement of -0.06 ± 1.2% BF. It should be noted that the research of Pineau et al. [20,21] used different ultrasound devices (US BOX, Lecoeur Electronique Co., Chuelles, France, and GEM, TEA Co., Vandoeuvre-les-Nancy, France) and prediction model than the BodyMetrix™ BX2000 and Jackson et al. formulas [7,8] used in the present study. However, other researchers have used the BX2000 in their validity studies [14,15,18,22,23]. Compared to skinfolds, Loenneke et al. [15] reported no significant difference but high *TE* in a small, mixed-sex group of college students, and Ulbricht et al. [18] reported no significant difference in the %BF estimation of 60 male military personnel despite some low correlations at the individual measurement sites. Recently, Smith-Ryan et al. [14] compared BX2000-estimated %BF using the 7-site Jackson and Pollock equation with a three compartment model that used the BOD POD to obtain Db and BIA to estimate total body water in a group of 47 overweight and obese adults. They found that, despite good reliability, the ultrasound significantly underestimated the %BF. In contrast, in a sample of 26 college students, Johnson et al. [22] reported significant correlations (*r* ≥ 0.86) and no significant differences between %BF estimates from the BX2000, ADP, and BIA. Finally, in a study of 70 euhydrated high school wrestlers, Utter and Hager [23] found excellent agreement between ultrasound and hydrodensitometry for the estimation of fat-free mass while skinfold significantly underpredicted this variable. Additionally, the *SEE* was less for ultrasound than skinfold, and these researchers concluded that ultrasound should be considered as an alternative method for estimating the fat-free mass of wrestlers.

There are several additional points to consider in the evaluation of the BodyMetrix™ BX2000 ultrasound. This is the first study to include more than 8 lean or average weight females. Smith-Ryan et al. [14] included 27 females in their study, but these were overweight and obese women. They found that the ultrasound method significantly underestimated %BF compared to a three component model, while we found a severe overestimation for lean females compared to the BOD POD. Also, while realizing that there is no true “gold standard” of body composition assessment, we assume that the BOD POD is a valid criterion method for this sample. Previous researchers have concluded that the BOD POD is a valid method for measuring %BF in female collegiate athletes [24,25]. Nevertheless, the %BF estimation from the BOD POD was significantly less than the estimates from both the ultrasound and skinfolds from both technicians; the possibility should be kept open that, despite being the criterion method in this study, the BOD POD could have underestimated the %BF of this particular sample. Using the Siri [9] conversion formula rather than a sex-specific or population-specific conversion formula could have also contributed to the difference between the Db-converted %BF estimate from the BOD POD and the algorithm-derived %BF from the ultrasound. However, the fat-free Db of female athletes has been reported to be 1.099 g/cc [6] which is nearly identical to the Siri-assumed fat-free Db of 1.100 g/cc; thus, it is unlikely that the use of the Siri formula rather than another conversion formula contributed substantially to the difference in %BF results. Finally, choosing the correct athletic type helps the Body View Professional software correctly process the ultrasound signal and identify the peak most likely associated with the fat-muscle boundary. There were a few instances in which the technicians overrode the software’s recommendation of an unrealistic reading and selected a different ultrasound peak as the measure of subcutaneous fat thickness. Peak selection is subjective, and choosing the wrong peak as the fat-muscle interface could result in substantial error. However, there is a scan mode that the technician can consult to verify the measurement when in doubt.

In summary, the BodyMetric™ BX2000 ultrasound device had excellent test-retest reliability as well as inter-rater reliability. The inter-rater reliability of the ultrasound was superior to the skinfold method. Overall, the ultrasound overpredicted %BF of collegiate athletes. However, this overprediction was more pronounced in the female athletes and of little practical significance in the male athletes. More research, such as a multicomponent validation study or a comparison with B-mode ultrasound, is warranted on this device, particularly given the paucity of research that includes large samples of female participants. Nevertheless, regardless of its questionable validity, given the excellent reliability, portability, and ease of use, A-mode ultrasound has promise as a method to assess change in %BF.

### Practical application

Whether it be tracking fat loss, muscle mass gains, or estimating ideal competitive weight, body composition assessment can serve as a beneficial tool for both coaches and athletes. Laboratory methods such as the BOD POD are costly; thus, more convenient and less expensive options like the skinfold method are typically used. However, as demonstrated in the current study, the inter-rater error of even experienced skinfold technicians can be substantial. When multiple observers, such as various strength coaches and athletic trainers, with varying levels of skill and experience are involved in assessments using skinfolds the inter-rater reliability will likely be poor. The ultrasound technique proved to have much higher inter-rater reliability than the skinfolds, making it more likely for multiple examiners to get similar results. More research is needed before this method can be recommended as a valid assessment of %BF in female athletes; however, our results combined with those of Utter and Hager [23] on high school wrestlers suggest that ultrasound is a valid alternative for estimating the %BF of male athletes. Regardless of its validity, we echo the sentiments of others who suggested that this device may be an effective tool for tracking changes in body composition due to its excellent reliability [14,15]. Combine this finding with the relatively inexpensive cost and ease of use, and the A-mode ultrasound could be a viable alternative for strength coaches and athletic trainers seeking to assess the body composition of their athletes.
